# Resolutions of N. Y. Homœopathic Union

**Published:** 1889-12

**Authors:** 


### RESOLUTIONS OF THE N. Y. HOMŒOPATHIC UNION.

The first regular meeting of the N. Y. Homœopathic Union for the season was held at the residence of Dr. E. Carleton,

Thursday evening, October 17th. The following resolutions were presented by Dr. Carleton and unanimously adopted by the Union:

WHEREAS, Our esteemed friend and colleague, Dr. Edward Bayard, has been called from the scene of his labors; therefore

*Resolved*, That in the death of this veteran physician we have sustained the loss of a leader who was ever true to the law of *similia* discovered by Hahnemann, zealous in the discharge of duty, eminently successful in healing the sick, a wise counselor, always courteous, a kind and valued friend to all who were so fortunate as to have his acquaintance.

*Resolved*, That while we deeply mourn his loss, we gratefully revere his memory and emulate his long and unselfish devotion to the promotion of the best interests of humanity.

*Resolved*, That these resolutions be spread upon our minutes, and that copies be sent to Dr. Bayard's family and to the medical press.

